# Reduced Levels of miR-342-5p in Plasma Are Associated With Worse Cognitive Evolution in Patients With Mild Alzheimer’s Disease

**DOI:** 10.3389/fnagi.2021.705989

**Published:** 2021-08-23

**Authors:** Farida Dakterzada, Iván David Benítez, Adriano Targa, Albert Lladó, Gerard Torres, Leila Romero, David de Gonzalo-Calvo, Anna Moncusí-Moix, Adria Tort-Merino, Raquel Huerto, Manuel Sánchez-de-la-Torre, Ferran Barbé, Gerard Piñol-Ripoll

**Affiliations:** ^1^Unitat Trastorns Cognitius, Clinical Neuroscience Research, Santa Maria University Hospital, IRBLleida, Lleida, Spain; ^2^Translational Research in Respiratory Medicine, Hospital Universitari Arnau de Vilanova-Santa Maria, IRBLleida, Lleida, Spain; ^3^Centro de Investigación Biomédica en Red de Enfermedades Respiratorias (CIBERES), Madrid, Spain; ^4^Alzheimer’s Disease and Other Cognitive Disorders Unit, Neurology Department, Hospital Clínic, Institut D’Investigacion Biomèdiques August Pi i Sunyer, Barcelona, Spain; ^5^Group of Precision Medicine in Chronic Diseases, Hospital Universitari Arnau de Vilanova-Santa Maria, IRBLleida, Lleida, Spain

**Keywords:** Alzheimer’s disease, cognitive decline, miRNA, miR-342-5p, biomarker

## Abstract

**Background:**

Progressive cognitive decline is the most relevant clinical symptom of Alzheimer’s disease (AD). However, the rate of cognitive decline is highly variable between patients. Synaptic deficits are the neuropathological event most correlated with cognitive impairment in AD. Considering the important role of microRNAs (miRNAs) in regulating synaptic plasticity, our objective was to identify the plasma miRNAs associated with the rate of cognitive decline in patients with mild AD.

**Methods:**

We analyzed 754 plasma miRNAs from 19 women diagnosed with mild AD using TaqMan low-density array cards. The patients were grouped based on the rate of decline in the MMSE score after 2 years [<4 points (*N* = 11) and ≥4 points (*N* = 8)]. The differentially expressed miRNAs between the two groups were validated in an independent cohort of men and women (*N* = 53) with mild AD using RT-qPCR.

**Results:**

In the discovery cohort, 17 miRNAs were differentially expressed according to the fold change between patients with faster declines in cognition and those with slower declines. miR-342-5p demonstrated differential expression between the groups and a good correlation with the rate of cognitive decline in the validation cohort (*r* = −0.28; *p* = 0.026). This miRNA had a lower expression level in patients who suffered from more severe decline than in those who were cognitively more stable after 2 years (*p* = 0.049).

**Conclusion:**

Lower levels of miR-342-5p in plasma were associated with faster cognitive decline in patients with mild AD after 2 years of follow-up.

## Introduction

Alzheimer’s disease (AD) is an irreversible, progressive brain disorder that gradually destroys memory and other thinking skills and, eventually, leads to complete dependency in daily life activities (Long and Holtzman, 2019). The rate of cognitive decline is highly variable among patients, with some having a faster course than others. Studies have demonstrated a strong association between the rate of cognitive decline and mortality in AD patients (Hui et al., 2003). In addition, understanding the physiopathological processes underlying this variability is highly important because of the great potential benefits for the development of effective therapeutic approaches.

Extracellular amyloid plaques (accumulation of amyloid-β (Aβ) protein) and intracellular neurofibrillary tangles (aggregations of hyperphosphorylated tau protein, P-tau) are two main pathological hallmarks of AD. Although both of these pathological characteristics are considered specific to AD, none of them have demonstrated a good correlation with the clinical symptoms (Jack et al., 2018). For example, the accumulation of Aβ plaques, known as the first pathological event of AD according to the “amyloid cascade hypothesis,” peaks at the asymptomatic stage of the disease (Selkoe and Hardy, 2016). It is widely accepted that neuronal injury, particularly synaptic loss, is the AD neuropathological alteration that most correlates with cognitive dysfunction (Jack et al., 2018; Colom-Cadena et al., 2020). The measurement of some proteins released in CSF, such as tau and neurofilament light chain, can be used to assess neurodegeneration and has shown a good correlation with cognitive decline (Jack et al., 2018; Preische et al., 2019). However, they are not specific to neuronal damage due to AD. In addition, the method for obtaining CSF is invasive, which limits its use for the concurrent monitoring of therapeutic trials and drug efficacy and for longitudinal studies where multiple lumbar punctures are needed. Therefore, searching for new biomarkers in the circulatory system that can predict the rate of cognitive decline and reveal neuropathological alterations specific to AD is of great importance.

MicroRNAs (miRNAs) are small (typically 22 nt in size) non-coding RNA molecules that regulate the activity of messenger RNA targets by binding to their specific binding sites located in the coding domain sequence or 3′-untranslated regions. This union suppresses the translation of the mRNA or induces its degradation (Brümmer and Hausser, 2014). miRNAs are present in tissues and bodily fluids and play important roles in a wide range of physiological and pathological processes, including AD (Cogswell et al., 2008; Hébert et al., 2008; Boissonneault et al., 2009). Circulatory miRNAs have shown high stability (Mitchell et al., 2008; Turchinovich et al., 2011), making them ideal biomarker targets. Increasing evidence indicates that miRNAs play a pivotal role in the regulation of synapses and synaptic plasticity (Hu and Li, 2017). Therefore, miRNAs are importantly involved in cognitive functions such as learning and memory (Wang et al., 2012). On the other hand, some studies have revealed that deregulation of several miRNAs contributes to synaptic and memory deficits in AD mouse models (Liu et al., 2017; Wang et al., 2018; Song et al., 2019).

In the present study, we aimed to detect and validate baseline circulating miRNAs that can be associated with the rate of cognitive decline in patients with AD after 2 years of follow-up. To this end, we selected a discovery cohort of women with mild AD and assessed their cognitive loss during a 2-year follow-up by the Mini-Mental State Examination (MMSE). The miRNAs present in the plasma of these patients were subjected to high-throughput miRNA expression profiling. Subsequently, the candidate miRNAs were validated in an independent cohort of men and women with mild AD via individual RT-qPCR methods.

## Materials and Methods

### Study Population

The subjects were prospectively recruited from a sample of outpatients who visited the Cognitive Disorders Unit at Hospital Universitari Santa Maria in Lleida and Hospital Clínic de Barcelona. The discovery cohort consisted of 19 women diagnosed with mild AD (MMSE score ≥20) and with abnormal Aβ42 levels (≤600 pg/mL) from Hospital Universitari Santa Maria in Lleida. The validation study consisted of 53 subjects with mild AD and abnormal Aβ42 levels from Hospital Universitari Santa Maria in Lleida (*N* = 41) and Hospital Clínic de Barcelona (*N* = 12). AD was diagnosed according to the clinical criteria of the National Institute on Ageing and Alzheimer’s disease Association (McKhann et al., 2011). Patients with cognitive impairment caused by other conditions, such as stroke, brain tumor, other neurodegenerative diseases, etc. were excluded from the study. We also excluded male patients from the discovery cohort to eliminate the sex effect in this small population.

Demographic data and general medical aspects such as hypertension, diabetes mellitus, hypercholesterolemia, stroke, depression, and APOE4 status were also evaluated in all subjects.

The cognitive evolution of the patients was measured by MMSE at baseline and after one and 2 years. The MMSE is a screening questionnaire for the detection of cognitive impairment (Folstein et al., 1975). It has 30 questions, and each question to be answered is scored with points, with a maximum possible score of 30 points. This questionnaire can be used to estimate the severity of cognitive impairment and to follow the course of cognitive changes in an individual over time. Although the rate of progression is variable among patients with mild AD, there is not any consensus regarding the definition of patients with fast decline versus slow decline. In a study by Stanley and collaborators, a 1.4 point/year (≈3 points/2 years) decline in MMSE was reported in patients aged >74 years (Stanley et al., 2019). Therefore, based on the rate of cognitive decline, we divided each cohort into two groups: patients who had lost less than 4 points (named slow decline in cognition, SDC) and those who had lost four or more points (named fast decline in cognition, FDC) after a 2-year follow-up.

### Sample Collection, RNA Extraction and Reverse Transcription

Blood samples were collected at baseline by venipuncture into EDTA-containing tubes between 8:00 and 10:00 A.M. in fasting condition. The samples were centrifuged at 2500 *g* for 10 min, and plasma was separated, aliquoted and stored at −80°C until use. Total RNA was extracted from plasma samples by using the *mir*Vana PARIS RNA and Native Protein Purification Kit (Cat. No. AM1556, Thermo Fisher Scientific) according to the manufacturer’s instructions. Isolation of total RNA instead of small RNAs was recommended by the manufacturer for miRNA expression profiling using miRNA arrays. Briefly, 300 μl plasma was added to an equal volume of 2× denaturing solution and then spiked with 10 μl of 100 pM synthetic cel-miR-39-3p (478293_mir, Thermo Fisher Scientific). The cel-miR-39-3p was added to the samples as an external control in order to monitor the RNA extraction efficiency. The phenol extraction was applied, and finally, total RNA was eluted in 40 μl of 95°C nuclease-free water following the recommended protocol. Two microlitres of RNA was reverse-transcribed to cDNA template and amplified using the TaqMan Advanced miRNA cDNA Synthesis Kit (Cat No. A25576, Applied Biosystems) and according to the corresponding user guide (publication number MAN0016122, revision C.0). The amplified cDNA product was stored at –20°C until use.

### Profiling of miRNAs Using TaqMan Low-Density Array (TLDA) Cards

The expression profiling of miRNAs in 19 plasma samples was carried out by loading a 1:10 dilution of amplified cDNA and TaqMan Fast Advanced Master Mix into two microarray cards (TaqMan Advanced miRNA Human A and B cards, Applied Biosystems), each containing 384 assays. The cards were run on a QuantStudio 7 Flex RT-PCR system (Life Technologies) and amplified based on the corresponding user guide (publication number MAN0016122, revision C.0). The threshold values were determined by QuantStudio software v-1.3. The data were processed with the Relative Quantification tool (powered by Thermo Fisher cloud), and their quality was evaluated based on the following criteria: (1) RT-PCR products were considered below the detection threshold and deleted if Ct ≥ 35 or if the Ct value were reported as “Undetected” and (2) RT-PCR products with an acceptable Ct range but an irregular amplification curve was censored. After evaluation of the quality of the raw data (Supplementary Figures 1, 2), they were normalized based on the mean-centering method, which is the gold standard when a high number of miRNAs are evaluated (Wylie et al., 2011; Faraldi et al., 2019).

### Validation of Differentially Expressed miRNAs by RT-qPCR

The differentially expressed miRNAs from the microarray experiment were validated in a new and independent cohort that consisted of 53 AD patients. RT-qPCR was carried out by using individual TaqMan Advanced miRNA Assays and TaqMan Fast Advanced Master Mix that were loaded on 384-well plates (Applied Biosystems). The samples were run in duplicate for each assay. We normalized these data using four endogenous controls (EC) (miR-103a-2-5p, miR-22-5p, miR-1301-3p, and miR-425-3p) and cel-miR-39 as an exogenous control. These ECs were shown to be stable in the plasma samples of controls and patients with or without pathophysiological changes in AD (Dakterzada et al., 2020).

### Target Analysis

We used TargetScan^1^ and miRDB^2^ tools to search for the possible target genes of the differentially expressed miRNAs. The biological targets of microRNAs in TargetScan are predicted by searching for the presence of sites that match the seed region of each miRNA (Bartel, 2009). In miRDB, miRNA targets are predicted from interactive modeling of miRNA binding and overexpression data (Liu and Wang, 2019).

### Ethics Approval

The Clinical Investigation Ethical Committee (CEIC P16/109) of Arnau de Vilanova University Hospital of Lleida approved this study for the discovery cohort. All patients included in the confirmatory cohort signed an internal written regulatory document stating that residual samples used for diagnostic procedures can be used for research studies.

### Statistical Analysis

Quantitative variables are shown as the mean (standard deviation) or median [interquartile range] according to the normality of the data. Absolute and relative frequencies were used to describe qualitative variables. We compared patient characteristics according to the study groups (FDC and SDC) in the discovery and validation cohorts. The *t*-test (or non-parametric Wilcoxon signed-rank test) was used to compare quantitative variables, and the chi-squared test was used for qualitative variables. The differences in miRNA expression between groups were evaluated using linear models for arrays (Ritchie et al., 2015). Given the age differences between study groups in the validation cohort, the linear models were age-adjusted in this cohort. The *p*-value threshold defining statistical differential expression was set at <0.05. miRNAs with significant difference between groups and a minimum fold change of 1.25 (or 0.8 for downregulated miRNAs) were considered as differentially expressed. All statistical analyses and data processing procedures were performed using R software, version 3.5.2 (Vienna, Austria).

## Results

### Patient Characteristics

The discovery and validation cohorts consisted of 19 and 53 patients with mild AD, respectively. In this regard, the pilot study consisted of 11 patients with SDC and 8 patients with FDC, while the validation cohort consisted of 32 SDC and 21 FDC patients. In the validation cohort, patients in the FDC group were older than those in the SDC group. There was no other significant difference regarding demographic data, comorbidities, AD core biomarker level or MMSE at baseline between patients included in each cohort (Table 1).

**TABLE 1:** Characteristics of the study population that participated in the discovery and validation cohorts.

	Discovery cohort			Validation cohort		
	SDC (−4,2]	FDC [−9,−4]	*p*-value	SDC (−4,5]	FDC [−16,−4]	*p*-value
	*N* = 11	*N* = 8		*N* = 32	*N* = 21	
**Demographic data**						
Sex, Female	11 (100%)	8 (100%)		10 (31.2%)	11 (52.4%)	0.211
Age	74.1 (5.43)	76.8 (6.02)	0.338	74.5 [71.5;80.0]	71.0 [66.1;75.0]	0.038
**Comorbidities**						
Hypertension	9 (81.8%)	4 (50.0%)	0.319	19 (59.4%)	10 (47.6%)	0.576
Stroke	2 (18.2%)	0 (0.00%)	0.485	1 (3.12%)	1 (4.76%)	0.999
Diabetes mellitus	2 (18.2%)	1 (12.5%)	1000	7 (21.9%)	4 (19.0%)	0.999
Dyslipidemia	4 (36.4%)	4 (50.0%)	0.658	12 (37.5%)	8 (38.1%)	0.999
Depression	5 (45.5%)	2 (25.0%)	0.633	10 (31%)	6 (28%)	0.861
APOE4 carrier, Yes	6 (54.5%)	5 (62.5%)	0.999	13 (54.2%)	10 (55.6%)	0.921
**Alzheimer’s parameters**						
Basal MMSE	21.3 (2.53)	24.5 (2.62)	0.017	23.0 (2.92)	23.8 (3.11)	0.348
1-year MMSE	22.2 (1.99)	22.2 (1.98)	0.942	22.4 (3.67)	19.7 (3.84)	0.016
2-year MMSE	19.8 (2.71)	18.6 (2.72)	0.36	22.1 (3.44)	16.7 (4.99)	<0.001
Aβ42	453 (116)	471 (86.0)	0.689	419 (100)	461 (106)	0.162
T-tau	601 [501;772]	594 [407;688]	0.563	430 [306;692]	618 [456;861]	0.094
P-tau	89.6 [76.8;118]	93.3 [78.8;106]	0.804	66.8 [51.5;94.6]	87.0 [75.7;110]	0.161

*SDC, slow decline in cognition; FDC, fast decline in cognition; MMSE, Mini-Mental State Examination; Aβ42, Amyloid beta 42; T-tau, Total tau protein; P-tau, Phosphorylated tau protein. P-values were calculated by comparing FDC and SDC patients using t-test (or non-parametric Wilcoxon signed-rank test) for continuous variables and chi-squared test for categorical variables.*

### Identification of miRNAs Related to the Rate of Cognitive Impairment (TLDA Experiment)

**FIGURE 1:**
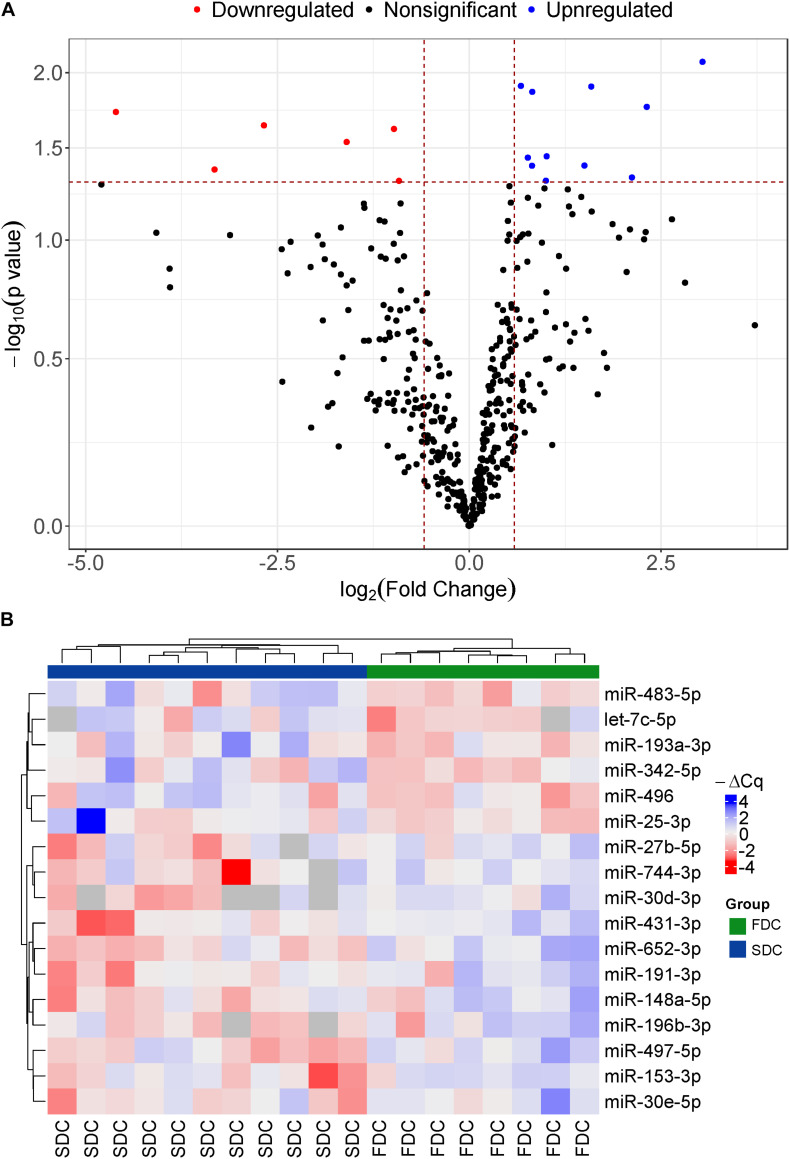
**(A)** Volcano plot of the distribution of 17 differentially expressed miRNAs at baseline between patients with FDC and SDC in the discovery study, mapping six downregulated miRNAs (red dots) and 11 upregulated miRNAs (Blue dots) in the FDC group; **(B)** Heatmap of all differentially expressed miRNAs at baseline between AD patients with FDC or SDC in the discovery cohort. Red is low expression and blue is high expression.

We identified 17 miRNAs that were differentially expressed between the two groups (Figure 1A). The expression profile of these 17 miRNAs was able to completely discriminate between the two groups of the study (Figure 1B). Among these 17 miRNAs, miR-25-3p (0.53 fold change), miR-496 (0.15 fold change), miR-342-5p (0.33 fold change), miR-193a-3p (0.1 fold change), miR-483-5p (0.5 fold change), and let-7c-5p (0.04 fold change) were downregulated in the FDC group (Figure 1A, red dots and Supplementary Table 1). Eleven miRNAs, including miR-30e-5p (2.01 fold change), miR-153-3p (1.69 fold change), miR-497-5p (1.76 fold change), miR-196b-3p (4.97 fold change), miR-148a-5p (2.83 fold change), miR-191-3p (1.76 fold change), miR-652-3p (1.59 fold change), miR-431-3p (3.01 fold change), miR-30d-3p (8.22 fold change), miR-744-3p (4.34 fold change), and miR-27b-5p (1.99 fold change), were upregulated in the FDC group (Figure 1A, blue dots and Supplementary Table 1). Furthermore, all 17 miRNAs had a good correlation with the rate of cognitive decline (Supplementary Table 1).

From these 17 miRNAs, 16 were selected for validation in an independent cohort. The sequences of all 17 miRNAs are shown in Supplementary Table 1. We eliminated the miRNA miR-193a-3p from the list of validation because it had a similar expression pattern between two groups except for 3 outlier patients in SDC group that had a lower expression than the other members of group.

### Validation of Differentially Expressed miRNAs

**FIGURE 2:**
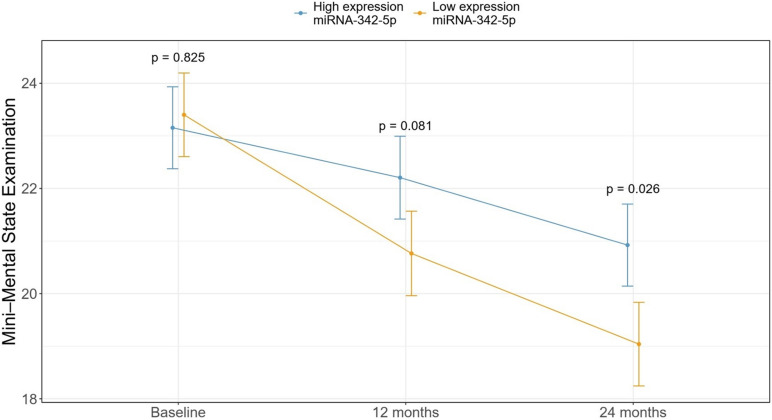
The association between the mean MMSE score in patients with high and low expression of miR-342-5p at baseline and after 1 and 2 years of follow-up. Patients with higher expression of miR-342-5p at baseline showed a slower cognitive deterioration after 2 years of follow-up compared with patients with low expression of this miRNA.

Individual RT-qPCR probes were used for validation of the 16-miRNA signature in an independent cohort of patients with mild AD (*N* = 53). After evaluation of the quality of the data (Supplementary Figure 3) and normalization, as explained in the Methods section, miR-342-5p showed significant differences in expression between the SDC and FDC groups (Supplementary Figure 4). Then, we dichotomized the patients into high and low expression of miR-342-5p groups, and our results revealed that patients with low expression of miR-342-5p had a worse cognitive evolution after 2 years of follow-up (*p* = 0.026) (Figure 2).

### Predicted Target Genes for miR-342-5p

We investigated the potential role of miR-342-5p in the pathological processes of memory loss in AD by searching through its predicted targets. Interestingly, not only proteins directly related to AD, such as beta-site amyloid precursor protein cleaving enzyme 1, microtubule associated protein 1A and tau tubulin kinase 1, but also the genes for many synaptic proteins were among targets that may be regulated by this miRNA (Table 2). Importantly, the ephrin A2 gene had the best target rank in miRDB and the second best target rank in TargetScan for miR-342-5p.

**TABLE 2:** Possible target genes of miR-342-5p found in miRDB and TargetScan databases that are associated with AD or synaptic plasticity.

Possible target	Gene symbol	Biological role related to synaptic plasticity and cognition (reference)	Database
**AD associated targets**			
Beta-site APP-cleaving enzyme 1	BACE 1	Breakage of amyloid precursor protein and production of Aβ protein (Cole and Vassar, 2007)	M & T
Tau tubulin kinase 1	TTBK1	Phosphorylation of tau protein (Taylor et al., 2019)	M & T
Microtubule associated protein 1A	MAP1A	Stabilization of microtubules (Fifre et al., 2006)	M & T
**Synapse associated targets**			
Ephrin A2 and A5	EFNA2 and A5	Regulation of contact-dependent cell communication (Chen et al., 2012)	M & T
Ephrin A3 and B1-B3	EFNA3 and B1-B3	Regulation of contact-dependent cell communication (Chen et al., 2012)	T
Eph receptor A8, A10, and B4	EPHA8, A10, and B4	Regulation of contact-dependent cell communication (Chen et al., 2012)	T
Eph receptor A1	EPHA1	Regulation of contact-dependent cell communication (Chen et al., 2012)	M
Synaptic Ras GTPase activating protein 1	SYNGPA1	Role in dendritic spine synapse maturation (Clement et al., 2012)	M & T
Insulin like growth factor 2	IGF2	Role in synaptogenesis and spine maturation (Pascual-Lucas et al., 2014)	M & T
Synaptophysin	SYP	Regulation of synaptic vesicle endocytosis (Kwon and Chapman, 2011)	T
Synaptopodin 2	SYNPO2	Role in dendritic spine stabilization (Yap et al., 2020)	T
Synaptosomal-associated protein, 91 kDa	SNAP91	Role in synaptic vesicle recycling (Vanlandingham et al., 2014)	M & T
Synaptosomal-associated protein, 47 kDa	SNAP47	Mediating synaptic vesicle fusion (Urbina et al., 2021)	T
NMDA receptor synaptonuclear signaling and neuronal migration factor	NSMF	Regulating synaptic stability and neuronal degeneration (Karpova et al., 2013)	M & T
Synaptic vesicle glycoprotein 2A and 2C	SV2A and 2C	Role in neurotransmission (Stout et al., 2019)	T
Neurogranin	NRGN	Role in post-synaptic signaling (Díez-Guerra, 2010)	T
Syntaxin 1	STX1	Mediating synaptic vesicle fusion (Lam et al., 2008)	M & T
Vesicle-associated membrane protein 2	VAMP2	Mediating synaptic vesicle fusion (Lam et al., 2008)	T
Vesicle-associated membrane protein 5	VAMP5	Mediating synaptic vesicle fusion (Lam et al., 2008)	M & T
Synaptotagmin 2, 5, 6, 7, 9, 11, and 17	SYT2, 5–7, 9, 11, and 17	Mediating synaptic vesicle fusion (Wu et al., 2020)	T
Synaptogyrin 3	SYNGR3	Regulation of neurotransmitter release (Raja et al., 2019)	T
Synaptojanin 1	SYNJ1	Role in synaptic vesicle recycling (Harris et al., 2000)	T
Synapsin 1	SYN1	Regulation of neurotransmitter release (Cesca et al., 2010)	T
Regulating synaptic membrane exocytosis 3	RIMS3	Regulation of synaptic vesicle fusion (Wang et al., 1997)	T
Bassoon presynaptic cytomatrix protein	BSN	Organizing neurotransmitter release site (Dresbach et al., 2003)	T
Glutamate ionotropic receptor NMDA type subunit 2A	GRIN2A	Neurotransmitter receptor (Paoletti and Neyton, 2007)	M
Neurexin 2	NRXN2	*Trans-*synaptic connector (Knight et al., 2011)	T
Complexin 1	CPLX1	Mediating synaptic vesicle fusion (Maximov et al., 2009)	T
Complexin 2	CPLX2	Mediating synaptic vesicle fusion (Maximov et al., 2009)	M & T

*AD, Alzheimer’s disease; M, miRDB; T, TargetScan.*

## Discussion

This study was designed to detect the association of miRNAs with the rate of cognitive decline measured by the MMSE in patients with mild AD using a hypothesis-free approach. We identified 17 miRNAs that were differentially expressed between women with AD who suffered from a faster cognitive decline over 2 years and those with slower cognitive decline. We validated these results in an independent cohort of men and women with AD. Our results revealed that miR-342-5p had a good correlation with the rate of cognitive decline, and the patients with lower expression levels of this miRNA had worse cognitive evolution after 2 years of follow-up.

Importantly, many differential miRNAs in the discovery study were previously associated with AD. For instance, miR-431-3p has been reported to prevent Aβ-mediated synaptic loss (Ross et al., 2018), and let-7c-5p, miR-483-5p, miR-342-5p, and miR-191-3p have been associated with AD in several studies (Takousis et al., 2019). In addition, miR-153-3p was reported to inhibit the expression of amyloid precursor protein (APP) (Liang et al., 2012; Long et al., 2012).

It is widely accepted that memory and cognitive impairment in AD primarily result from synaptic failure. Gene array experiments have shown alterations in genes involved in neurotransmitter receptors and receptor trafficking, synaptic vesicle trafficking and release, cell adhesion regulating synaptic stability, post-synaptic density scaffolding, and neuromodulatory systems in the early stages of AD (Scheff et al., 2007; Chang et al., 2012; Berchtold et al., 2013). Although the association of miRNAs with AD has been widely studied, there is scarce information about the miRNAs related to the rate of cognitive impairment and their role in the processes that underlie cognitive decline in these patients. To date, some studies have been conducted to determine the miRNAs important for the regulation of synaptic structure and function and to study their dysregulation and their effect on cognitive impairment in animal models of AD (Liu et al., 2017; Wang et al., 2018; Song et al., 2019; Barros-Viegas et al., 2020). For example, Song et al. (2019) found that upregulation of miR-30b causes synaptic and cognitive deficits in 5XFAD APP transgenic mice. miR-30b targets molecules such as ephrin type-B receptor 2, sirtuin1, and glutamate receptor subunit 2 that are important for maintaining synaptic integrity. These investigators observed that WT mice treated with miR-30b showed impaired spatial learning and memory retention measured by the Morris water maze and novel object recognition tests (Song et al., 2019). In a study by Barros-Viegas and collaborators, overexpression of miR-31-5p in a 3×Tg-AD model resulted in a better performance of animals in the T-maze, novel object recognition and Barnes maze, which were used for assessing spatial memory and short-term and long-term memory, respectively (Barros-Viegas et al., 2020).

To our knowledge, this is the first study in which the association of miRNAs has been evaluated with cognitive evolution in patients with mild AD. In a targeted study by Tan et al. (2014) the serum levels of several miRNAs related to AD were evaluated in patients with AD and healthy controls. They observed that serum levels of miR-125b had a negative correlation with MMSE score in AD patients (Tan et al., 2014). Wiedrick and collaborators assessed the correlation of the CSF levels of 14 miRNAs that were differentially expressed between AD and controls with MMSE. They observed that in these profiles, miR-193a-5p showed a higher correlation with MMSE (Wiedrick et al., 2019). However, in contrast to our study, in none of these studies assessed the association of miRNAs with the cognitive evolution of AD during a given time of follow-up. In a study by Mengel-From et al. (2018) the association between plasma miRNAs and MMSE and Cognitive Composite Score (CCS) was evaluated in healthy aged twins followed-up for 10 years. They observed that miR-151a-3p, miR-212-3p, and miR-1274b were associated with CCS in both the individual and paired analyses. miR-548c-3p, miR-539-5p, miR-532-3p, miR-369-3p, miR-548a-3p, and miR-27a-5p were associated with the MMSE score (Mengel-From et al., 2018).

In the present study, we validated the differential expression of plasma miR-342-5p between AD patients with FDC and SDC. This miRNA had a good correlation with the rate of cognitive decline evaluated by the MMSE. Interestingly, we found the genes for many synaptic proteins, including ephrin A2, syntaxin, synaptotagmin, synaptojanin, and neurogranin, among possible targets of miR-342-5p. Some of these targets, such as neurogranin, have been shown to have a good correlation with the rate of cognitive decline in patients with AD (Portelius et al., 2015; Headley et al., 2018). Therefore, it is possible that miR-342-5p plays a part in the cognitive alteration of AD via the regulation of some synaptic genes. However, this hypothesis should be tested in cellular experiments and animal models of AD.

miR-342-5p was reported to regulate the proliferation and differentiation of neuronal stem cells (Gao et al., 2017). Sun et al. (2014) reported higher levels of miR-342-5p in APP/PS1, PS1DE9, and PS1-M146 V mouse models than in the wild-type mouse brain and suggested that miR-342-5p plays a role in AD axonopathy by hampering the function of the axon initial segment via downregulation of ankyrin G. Although it is challenging to compare our results with the results of other studies in which different tissues and species and study designs were used, our result is not in accordance with the results presented by Sun et al. (2014) because we detected higher levels of miR-342-5p in patients who were cognitively more stable than those who were suffering from a more severe decline. However, our results are in agreement with the study by Lugli et al. (2015), who reported downregulation of miR-342-5p in plasma exosomal samples of patients with AD compared with non-demented controls.

This study has several strengths. We identified and validated miRNAs related to the rate of cognitive evolution in two independent cohorts. We only included mild AD patients with pathological levels of Aβ42 in both cohorts to assure that assessed cognitive decline is due to AD. Furthermore, we evaluated a high number of miRNAs in the discovery study to identify all possible candidates. Finally, the cognitive alterations of the patients were followed-up for 2 years, while in previous studies (Tan et al., 2014; Wiedrick et al., 2019), the association of miRNAs was assessed with the cognitive status of the patients at baseline.

This study has some limitations. First, the discovery cohort consisted of only female participants; however, we included male subjects in the validation cohort to overcome the bias that may have been caused by this issue. Second, the number of participants in the both discovery and validation cohorts was small, which may have affected the final result of our study. Third, there was a significant difference regarding baseline MMSE scores between FDC and SDC groups in the discovery cohort. Finally, from 16 differential miRNAs selected for validation, we only validated one miRNA. All aforementioned limitations and variabilities related to the normalization method and analytical platforms between two cohorts may have caused this inconsistency. Therefore, this result does not rule out the importance of other differential miRNAs in the cognitive evolution of AD.

To our knowledge, this is the first study seeking to identify miRNAs related to the rate of cognitive decline in patients with mild AD among a large profile of miRNAs. We detected 17 miRNAs that were able to perfectly separate AD patients with FDC from those with SDC. From this panel, we validated that miRNA miR-342-5p was associated with the rate of cognitive decline in an independent cohort, suggesting that uncovering the role of miRNAs in cognition may be of interest in seeking new biomarkers and furthering our understanding of the neuropathological processes underlying cognitive decline in AD.

## Data Availability Statement

The raw data supporting the conclusions of this article will be made available by the authors, without undue reservation.

## Ethics Statement

The studies involving human participants were reviewed and approved by the Clinical Investigation Ethical Committee (CEIC P16/109) of Arnau de Vilanova University Hospital of Lleida. The patients/participants provided their written informed consent to participate in this study.

## Author Contributions

FD, ID, AT, and GP-R designed the study. FD and AM-M carried out the experiments. ID, AT, GP-R, AL, AT-M, FB, and FD analyzed the data. FD, ID, AT, AL, GT, LR, DG-C, AT-M, RH, MS-T, FB, and GP-R interpreted the data. FD, ID, and GP-R wrote the manuscript draft. All authors revised the manuscript and approved it for submission.

## Conflict of Interest

DG-C has filed a patent on miRNAs as biomarkers. The remaining authors declare that the research was conducted in the absence of any commercial or financial relationships that could be construed as a potential conflict of interest.

## Publisher’s Note

All claims expressed in this article are solely those of the authors and do not necessarily represent those of their affiliated organizations, or those of the publisher, the editors and the reviewers. Any product that may be evaluated in this article, or claim that may be made by its manufacturer, is not guaranteed or endorsed by the publisher.

## Footnotes

1
http://www.targetscan.org/vert_72/

2
www.mirdb.org
