# Supplementary material for: Reduced Levels of miR-342-5p in Plasma Are Associated With Worse Cognitive Evolution in Patients With Mild Alzheimer’s Disease
Source: Front Aging Neurosci. 2021 Aug 23;13:705989. doi: 10.3389/fnagi.2021.705989 (PMC8421031; doi:10.3389/fnagi.2021.705989)

**Supplementary data**

**Supplementary Figure 1** Quality control of TaqMan Low Density Array (TLDA) determinations. Number of determinations/missings.


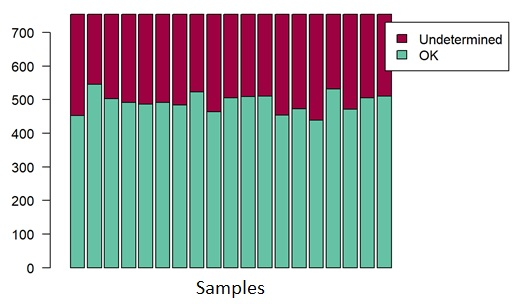


**Supplementary Figure 2** Distribution of miRNAs overall expression in TLDA cards. The total expression of miRNAs was homogenous between study subjects in the discovery cohort. A Ct cut-off of ≤ 35 was defined as acceptable.


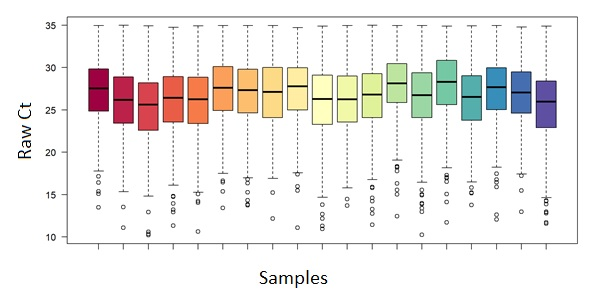


**Supplementary Table 1** miRNAs expressed differentially between FDC and SDC groups in the discovery cohort.

| **miRNA** | **Sequence** | **Fold change** | **P-value** | **Correlation with MMSE score at 2 years** |
| --- | --- | --- | --- | --- |
| miR-30d-3p | UGUAAACAUCCCCGACUGGAAG | 8.22 | 0.0083 | 0.62 |
| miR-652-3p | AAUGGCGCCACUAGGGUUGUG | 1.59 | 0.0124 | 0.78 |
| miR-431-3p | CAGGUCGUCUUGCAGGGCUUCU | 3.01 | 0.0126 | 0.64 |
| miR-497-5p | CAGCAGCACACUGUGGUUUGU | 1.76 | 0.0137 | 0.38 |
| miR-196b-3p | UCGACAGCACGACACUGCCUUC | 4.97 | 0.0173 | 0.58 |
| hsa-let-7c-5p | UGAGGUAGUAGGUUGUAUGGUU | 0.04 | 0.0187 | -0.53 |
| miR-496 | UGAGUAUUACAUGGCCAAUCUC | 0.15 | 0.0229 | -0.59 |
| miR-483-5p | AAGACGGGAGGAAAGAAGGGAG | 0.50 | 0.0241 | -0.49 |
| miR-342-5p | AGGGGUGCUAUCUGUGAUUGA | 0.33 | 0.0291 | -0.23 |
| miR-30e-5p | UGUAAACAUCCUUGACUGGAAG | 2.01 | 0.0355 | 0.41 |
| miR-153-3p | UUGCAUAGUCACAAAAGUGAUC | 1.69 | 0.0362 | 0.42 |
| miR-148a-5p | AAAGUUCUGAGACACUCCGACU | 2.83 | 0.0403 | 0.52 |
| miR-191-3p | GCUGCGCUUGGAUUUCGUCCCC | 1.76 | 0.0404 | 0.49 |
| miR-193a-3p | AACUGGCCUACAAAGUCCCAGU | 0.10 | 0.0424 | -0.52 |
| miR-744-3p | CUGUUGCCACUAACCUCAACCU | 4.34 | 0.0471 | 0.45 |
| miR-27b-5p | AGAGCUUAGCUGAUUGGUGAAC | 1.99 | 0.0491 | 0.41 |
| miR-25-3p | CAUUGCACUUGUCUCGGUCUGA | 0.53 | 0.0492 | -0.51 |

**Supplementary Figure 3** Distribution of miRNAs overall expression in the validation cohort. The total expression of miRNAs was homogenous between study subjects in the validation cohort. A Ct cut-off of ≤ 35 was defined as acceptable


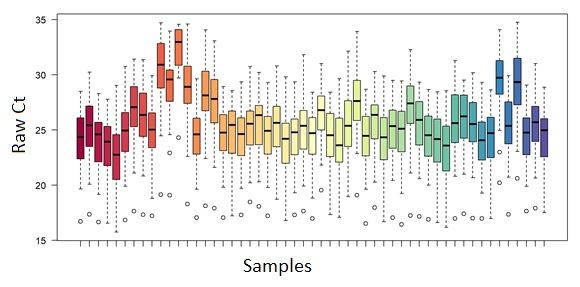


**Supplementary Figure 4** Box plot diagram of the relative expression of miR-342-5p among AD patients with FDC and SDC measured by qRT-PCR. The miR-342-5p level was higher in plasma of patients with SDC than those with FDC (p=0.049).


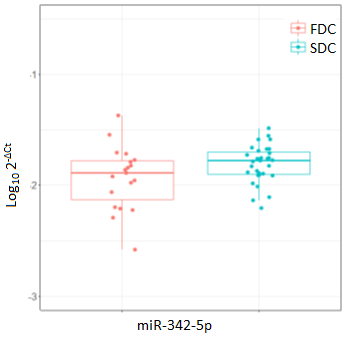

Supplement: Supplementary file 1 [file Data_Sheet_1.docx]
